# Implementation of an SfM-MVS-based photogrammetry approach for detailed 3D reconstruction of plants

**DOI:** 10.1186/s13007-025-01445-x

**Published:** 2025-10-09

**Authors:** Jiří Mach, Zdeněk Svatý, Ondřej Šoupa, Luboš Nouzovský, Martin Halecký

**Affiliations:** 1 Department of Biotechnology, Faculty of Food and Biochemical Technology, University of Chemistry and Technology in Prague, Technická 5, Prague 6, 166 28 Czech Republic; 2 Department of Forensic Experts in Transportation, Faculty of Transportation Sciences, Czech Technical University in Prague, Horská 2040/3, Prague 2, 128 03 Czech Republic

**Keywords:** Close-range photogrammetry, SfM-MVS-based data processing, 3D reconstruction, Plant phenotyping, Morphological traits, Precision agriculture

## Abstract

In recent years, non-destructive and non-invasive methods for 3D plant reconstruction have gained increasing importance in plant phenotyping. Morphological traits reflect the physiological status of a plant and serve as key indicators for precision agriculture, crop protection, and food quality assessment. Accurate and efficient 3D modelling enables objective and repeatable monitoring of plant development and health, thus supporting data-driven decision-making in agricultural and food research. This study presents a novel, cost-effective, and flexible photogrammetric apparatus for the routine analysis of plant morphological traits under controlled laboratory conditions. Existing systems often rely on expensive instrumentation and provide limited adaptability, whereas the platform described here combines affordability with high precision and robustness. A key innovation is the use of a robotic arm to control an industrial RGB camera, providing substantial flexibility in image acquisition. This mobility ensures comprehensive coverage of plants of different sizes and architectures while minimising occlusions. Another distinctive feature is the implementation of an optimised parameter tweak in the photogrammetric pipeline, which markedly improves the reconstruction of thin and delicate plant parts such as leaves, petioles, and fine stems. In combination with optimised acquisition parameters, including an exposure time of 50 milliseconds, a tweak value of 0.9, and a camera-to-object distance of 16 centimetres, the system achieves consistent model fidelity across diverse plant structures. Efficiency was further enhanced through automation and an optimised scanning procedure. Comparative testing showed that using a larger number of camera positions with fewer frames per position improved throughput, with the best configuration consisting of three height levels and 40 frames each. These improvements reduced the processing time by 75%, decreasing the average scan duration from 8 min to only 2.7 min per plant, while maintaining accuracy and reliability. Overall, the developed apparatus constitutes a reliable and low-cost solution that integrates robotic-assisted flexibility, improved reconstruction through the parameter tweak, and markedly reduced scanning time. The combination of precision, affordability, and efficiency makes the system competitive with existing approaches and, due to its accessibility and detailed methodological description, provides a distinctive contribution to the phenotyping community.

## Background

Recent advances in computational power, combined with the widespread availability of digital cameras, have driven a significant paradigm shift in photogrammetric techniques. This progress has enabled the development of advanced methods such as structure from motion (SfM) and multi-view stereo (MVS), which have been increasingly applied in precision agriculture, particularly for plant phenotyping. These techniques provide non-invasive and non-destructive means to accurately capture a broad range of plant traits through detailed 3D point cloud reconstruction. Moreover, they offer promising potential for the future automation of phenotyping workflows [1]. The methodology of precise plant phenotyping incorporates not only the analysis of plant vitality and prosperity (vegetation indices) but also the characteristics of the plant architecture, namely volume, area, biomass density along the main axis of the plant, leaf inclination, and configuration [2, 3, 4]. In contemporary scientific disciplines such as plant and plant pathology, a non-destructive approach to the study of morphological features has emerged as the prevailing standard [5, 6, 7]. The use of such a methodis widely embraced within the scientific community, which signifies its status as a contemporary paradigm in the scientific study of plants.

The key indicators enumerated above are typically pivotal in the study of the interaction between a plant and an external factor, which may be an abiotic or biotic agent. As the global average temperature increases, pressure is placed on plant breeders to develop cultivars that are resistant to drought and other adverse soil conditions [8, 9]. These conditions can cause stress to plants, which can have a negative impact on their development and prosperity. High salt concentrations in soil have also been demonstrated to be detrimental to proper metabolic processes within the plant body, as well as to the development of the plant body in general [10]. In contrast, factors that have been demonstrated or have the potential to exert a favourable influence on plant growth are worthy of mention. These biopreparations are either in conventional use or are undergoing development and laboratory testing. They are composed of cellular or cell-free mixtures of microorganisms and/or their biologically active metabolites. The function of biostimulants is to promote plant growth, either directly or indirectly. These preparations have the ability to increase the bioavailability of crucial soil elements, such as phosphorus [11, 12, 13]. It is evident that auxin phytohormone microbial producers also belong to this category of biopreparations, which have been demonstrated to exert a positive influence on plant metabolism [14]. The extensive list of biopreparations includes the widely used Polyversum^®^, which is derived from the mycoparasitic oomycete *Pythium oligandrum* and is effective in the elimination of fungal diseases [15]. The development of potential biopreparations is frequently conducted within a laboratory setting, with subsequent testing being conducted directly on economically significant crops, including but not limited to barley, wheat, maize, rice, and soybean [16]. All the abiotic and biotic factors mentioned above, which exert a negative or positive influence on plants, have one thing in common, namely the initiation of a morphological response of the plant body by their presence. In the following section, the various technological approaches currently employed or with potential for application are comprehensively delineated. The focus will be on non-destructive methods for investigating the effects of a factor on the morphological characteristics of the affected plant.

In accordance with the principle of spatial data acquisition, available 3D reconstruction technologies can be classified into two distinct groups: active and passive systems. The former relies on the emission of active radiation and the subsequent measurement of its reflection from the object surface. A terrestrial, mobile, or airborne scanner emits a laser beam for the purpose of distance sensing [17, 18, 19, 20, 21, 22]. Time-of-flight depth cameras utilise structured light to determine the distance to the surface of an object by measuring the time delay between light emission and its reflection back to the sensor [23, 24]. High-precision X-ray tomography is a reliable method to scan detailed objects; however, it is relatively expensive [3, 25]. The second group covers passive methods that do not rely on light emission. Instead, these methods use ambient electromagnetic radiation that is naturally emitted by the sun or other artificial light source. Photogrammetry is a process that uses images captured from multiple angles; however, it is susceptible to variations in lighting conditions and the texture of the object in question [26].

In the domain of photogrammetric analysis, two principal strategies are employed for the acquisition of images. The first method is based on moving-camera systems with static-object, where the object remains stationary while the imaging device moves around it [27, 28]. Second, static-camera moving-object systems are considered, in which the camera remains stationary while the object is rotated or translated in front of it [29]. The use of both configurations in close-range photogrammetry is contingent upon the specific experimental setup, the measured object properties, and the spatial constraints inherent to the imaging environment. The combination of the aforementioned methods is indeed feasible. In such cases, the camera is usually attached to the moving component [30, 31]. The control software deploys the arm in a series of predetermined positions to achieve the desired image configuration. At each position, the camera remains for a predefined period, during which a rotating object is sensed. The process subsequently transitions to the subsequent position, and this sequence is repeated. The acquired images are processed using the structure-from-motion (SfM) and multi-view stereo (MVS) algorithms [32, 33]. SfM is a process that facilitates the recovery of both camera poses and a sparse 3D point cloud. This is achieved by detecting and matching key features on the object surface (keypoints) across overlapping images. In the context of close-range photogrammetry, particularly in the field of plant science, the process of photo alignment is frequently used. It is often facilitated by the strategic placement of an object within the scene that exhibits a distinctive and unique multicoloured texture. Examples of such objects include a multicoloured cube, ball, or plate [34]. Matched features, termed tie points, are utilised in a subsequent bundle adjustment procedure, which optimise both interior camera parameters and exterior orientations by minimising reprojection error. This stage, known as photo alignment, results in a geometrically consistent configuration of the image set and an initial sparse reconstruction of the scene. Following successful alignment, MVS algorithms are employed to densify the point cloud, thereby computing dense pixel-wise correspondences between images to generate a detailed and accurate surface model. This workflow is a common feature of commercial photogrammetric software, such as Pix4Dmapper, Metashape, ContexCapture or RealityCapture, which automates the majority of these steps while allowing for fine user control over parameters influencing reconstruction quality [35, 36]. When targeting small-scale, highly detailed biological samples, such as horticultural plants, it is imperative to employ high-resolution image acquisition techniques. This involves employing diffuse and uniform lighting to minimise shadows and reflections, applying background separation techniques such as chroma keying or the use of plain, low-texture surfaces, and carefully calibrating both interior and exterior camera parameters to reduce distortion and enhance spatial accuracy.

At present, a considerable number of research institutes are engaged in the study of close-range photogrammetry as applied to plant science. The focus of this field of study is the analysis of the aerial parts of plants. The objective is to generate the most detailed model from which the position, orientation, and distribution of the leaf can be accurately determined [37]. The leaf area index (LAI) is defined as the ground area covered by the plant canopy projected onto the soil surface. It serves as a critical qualitative and quantitative descriptor of morphological traits [38]. LAI plays a central role in the optimisation of (bio)preparation dosages for plant treatments [39, 40]. Furthermore, the architecture of the root system of the plant can be investigated in detail [41, 42, 43]. To ensure the effective and consistent implementation of photogrammetric scanning systems, it is imperative to meticulously calibrate and set a number of parameters. These include determining the interior and exterior orientations of the cameras, the prevailing lighting conditions, the distance at which the scanning is conducted, and the evaluation pipeline used for the generation of 3D models [1, 44, 45, 46]. For example, the Perceptron v5 laser scanner is often used as a reference device to evaluate the reliability, accuracy, and usability of photogrammetric systems [33, 47]. The primary aim of the previously mentioned studies is to expand the portfolio of precise and cost-effective photogrammetric platforms suitable for high-throughput plant phenotyping applications. This study further contributes to this goal by developing and evaluating an additional low-cost photogrammetric platform.

The objective of the present study is to extend the functionality of the existing multifunctional scanning apparatus [48]. The original methodology of plant reconstruction, which relies on the use of a 3D laser scanner, is to be superseded by a more appropriate technique (for this experimental setup). The latter will be subject to an SfM-MVS-based photogrammetric analysis. This will be carried out using an industrial RGB camera mounted on a robotic arm, a turntable, and additional LED illumination. The subsequent analysis of the acquired images will be undertaken with the objective of generating the most accurate 3D model. The subject of interest will be the calibration of the camera and the optimisation and appropriate adjustment of the scanning parameters (e.g., exposure time and scanning distance). The primary evaluation criterion will be the quality and detail of the generated model for each of the three plant species, as indicated by the total area and volume of the digital biomass. Subsequently, the selected statistical indicators of the quality of the spatial reconstruction are assessed. The apparatus should ideally be capable of producing an image of the plant that allows the creation of a comprehensive spatial model while preserving significant detail. These features will be of particular importance in future research, where the focus will be on a non-destructive way to determine the positive effect of a biocontrol agent or the negative effect of abiotic and biotic stressors on plant growth. Consequently, the accuracy and reliability of the scanning process and back reconstruction will play a pivotal role in this subsequent phase of research.

## Methods

The subsequent chapter addresses the enhancement of the functionality of the multifunctional scanning apparatus that was previously developed and described [48]. This enhancement is achieved through the application of the photogrammetric method, which allows improved data acquisition and more detailed 3D reconstruction. Specifically, it provides a comprehensive description of the necessary components, including a detailed description of the object to be scanned, the optimisation procedure within the photogrammetric analysis and computational complexity, and the method of evaluation and interpretation of the resulting data.

### Photogrammetric system setup

#### Apparatus components

**Fig. 1:**
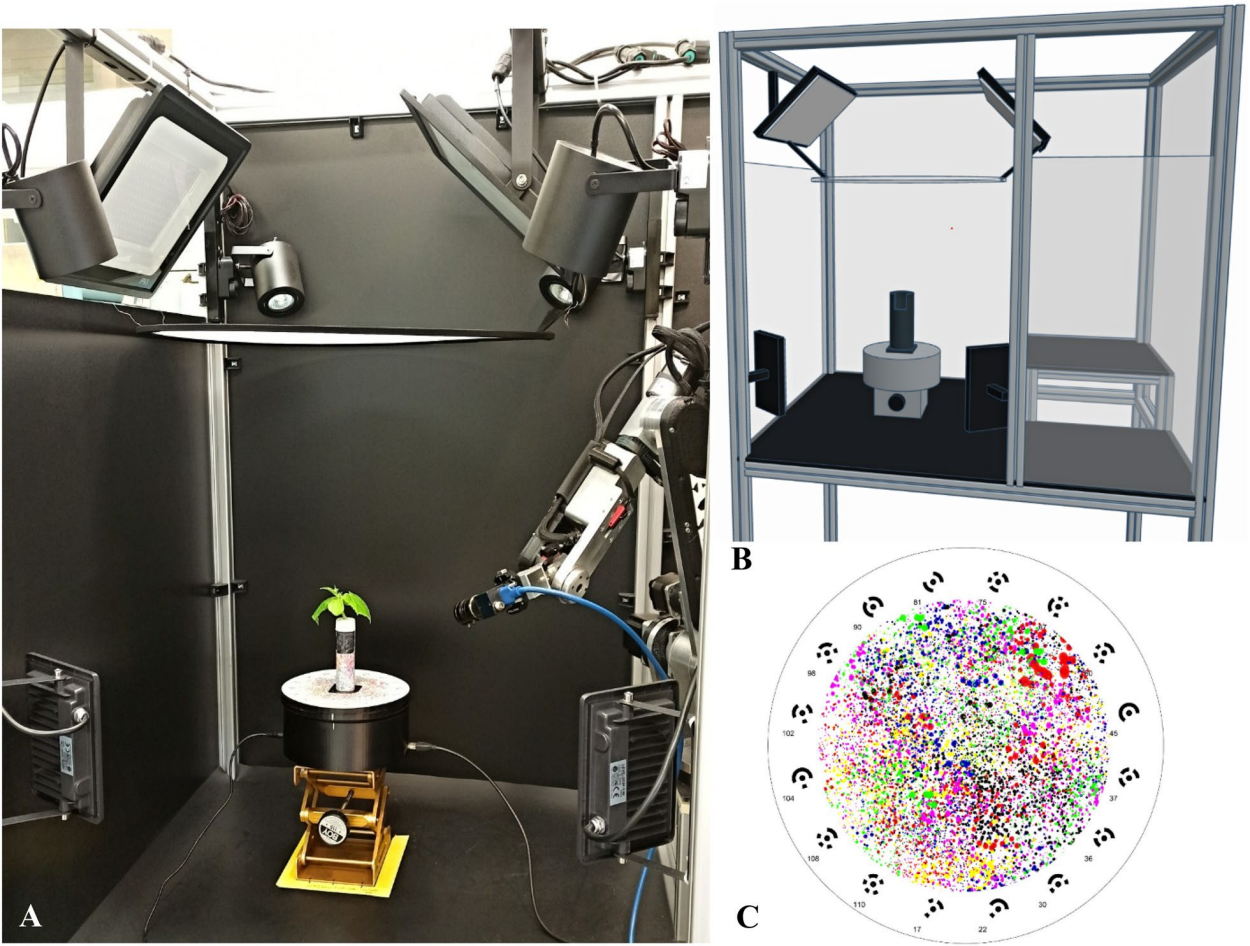
Representation of the scanning apparatus under development: A – The scanning photogrammetric apparatus consists of the following main components: a robotic arm, an RGB camera, a motorised turntable, and additional LED illumination with a diffusion membrane; B – The illustrative CAD model; and C – The turntable surface with a coloured splatter pattern and 16 control points

The functionality of the multifunctional scanning apparatus was further enhanced by integrating the capability to perform semi-automatic photogrammetric reconstruction of scanned samples, thereby broadening its range of applications. The enhancement was attributed to the addition of auxiliary illumination (see Figs. 1A and B). The lighting system consisted of two 200 W overhead LED reflectors and two 30 W side LED LEVE supplementary reflectors, providing a combined light intensity of approximately 500 µmol/m²/s across the scene. With this intensity value, the surface of the object was evenly illuminated. Diffused and sufficient illumination was crucial to ensure correct and accurate photogrammetric reconstruction. It also plays a key role in achieving a high-quality 3D model that preserves morphological details and structural features. The homogenisation of object illumination was ensured by a photographic diffusion panel placed above the plant, with emphasis on minimising sharp shadows and uneven light exposure across the scene. Additionally, two undiffused 30 W LED panels provided the sharp side lighting necessary for accurate QR code scanning of the cuvette. The second significant component used in the process was a MER2-1220-32U3C camera, which was equipped with an LCM-12MP-06MM-F2.4-1.7-ND1 lens (Daheng Imaging, China). The camera generated 24-bit colour (RGB) images in JPEG format, which were subsequently used as input for photogrammetric processing to reconstruct 3D models. The technical specifications of the imaging system, including the camera and lens, are summarised in Table 1. The camera was mounted on an AR4 robotic arm (Annin Robotics, USA) with 6 degrees of freedom to ensure precise positioning during imaging. To enable semi-automatic scanning, the scanned object was always placed in a custom-made holder attached to the turntable. Both components were printed from ABS filaments via the standard fused deposition modelling method with an i3 MK3S 3D printer (Original Prusa, Czech Republic). The turntable was driven by an NEMA17 stepper motor with a TMC2130 driver, and control was mediated by an ArduinoUNO platform with an ATmega328 microcontroller. The surface of the object holder and the top of the turntable were covered with a custom-made randomly coloured splatter texture, forming a unique pattern specifically designed for this setup (see Fig. 1 – C). This improved the strength and quality of the alignment of the captured images during the 3D model generation procedure. In addition, sixteen control points (12-bit coded circular targets) were placed on the surface of the turntable, allowing control of the spatial reconstruction accuracy and definition of the scale and coordinate system of the resulting digital model. The original system [48], priced at 8,790 EUR (excluding the 3D scanner and dual-axis turntable), was upgraded by adding a camera and lens costing 388 EUR, bringing the total hardware cost to 9,178 EUR. However, this amount does not include the software licence for Agisoft Metashape, which is priced at 540 EUR for the Professional Educational version (3,460 EUR for the full Professional (non-educational) edition).

**Table 1:** List of technical parameters of industrial RGB camera and lens (taken from the manufacturer)

	Description or value
**Camera MER2-1220-32U3C parameters**	
Communication interface	USB3
Resolution	4024 × 3036 (12.2 MPx)
Frame rate	32 fps
Pixel size	1.85 μm
Sensor	1/1.7’’ CMOS
Bit depth of the pixels	8bit, 12bit
Pixel data format	BayerRG8, BayerRG12
Weight	65 g
Dimensions	29 × 29 × 29 mm
Price*	252 EUR
**Lens LCM-12MP-06MM-F2.4-1.7-ND1 parameters**	
Lens mount	C-mount
Opticalresolution	12 Mpx
Image format	1/1.7’’
Focal length	6 mm
IR corrected	None
Aperture (min)	F2.4
Iris	Manual
Working distance	100 mm - infinity
Lens dimensions	29.8 × 37.72 mm
Distortion	< 0.05%
Price*	136 EUR

* The rounded prices include VAT and correspond to the exchange rate of 18 July 2025 (24.625 CZK per 1 EUR, source: CNB in Prague).

#### Scanned objects

The following model plants were selected for optimisation and verification of the reliability of the developed photogrammetric approach: *Cucumis sativus* L. (cucumber), *Solanum lycopersicum* L. (tomato), and *Lactuca sativa* L. var. *capitata* L. (lettuce). Henceforth, only the Latin plant nomenclature will be used throughout the remainder of this text. The selection of these plants was driven by their distinctive morphological characteristics, namely their small, thin, and flat features, which serve as a suitable model for the development of an accurate and reliable photogrammetric method. All three of these plants are significant agricultural and food crops. The plants were grown under constant temperature conditions of 25 °C and a light intensity of 160 µmol/m^2^/s, characterised by a long photoperiod of 16 h of light and 8 h of darkness. A horticultural substrate was used, placed in 30 ml of PP Sterilin cuvettes with a hole in the cap. The use of the cuvette as a cultivation vessel was crucial, as the holder on the rotary turntable was specifically designed to accommodate it, thus facilitating the handling of the plants. A further advantage of this configuration was the ease with which the camera could access the underside of the rosette leaves, thus facilitating the acquisition of the data necessary to generate a full high-quality 3D model. Finally, it also enables the use of an automatic boundary definition for spatial reconstruction, lowering computational and time demands. Scanning was conducted on three-week-old plants.

#### Spatial configuration of the scanning process

**Fig. 2:**
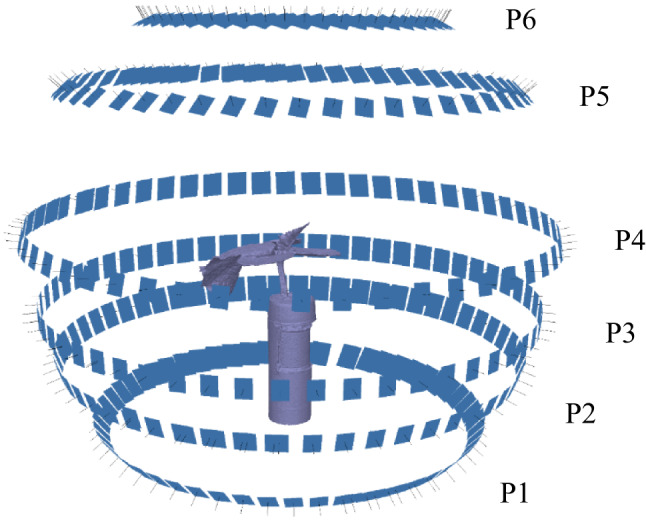
Illustrative example of the predefined image acquisition scheme. The blue rectangles indicate the imaging configuration across six height levels (P1–P6), with 6 × 60 images in total

The scanning process was conducted through a static-camera moving-object system, with the camera sequentially positioned at six distinct height levels. The procedure was characterised by the use of discrete, robotically controlled viewpoints, which enabled the autonomous acquisition of multiple images. The movement of the robotic arm, which is predefined in the basic settings, ensures that the predefined image configuration is achieved (height, distance from the object, angle of the camera) at each of the six locations. During the image acquisition process, the camera was positioned at predefined height levels and held in place long enough to capture up to 60 images at each level (see Fig. 2). In particular, the camera did not initiate capture during movement between positions. The scanning process was automatically initiated by a predefined position of the turntable with the object attached to the initial position. This was facilitated by the Hall sensor, which detected the presence of a magnet on the rotating top of the turntable. During imaging, the rotation speed was continuous, with an angular rotation speed of 6°/s (~ 0.105 rad/s). The speed was set to minimise any movement of the scanned object.

#### Acquisition and data processing

**Fig. 3:**
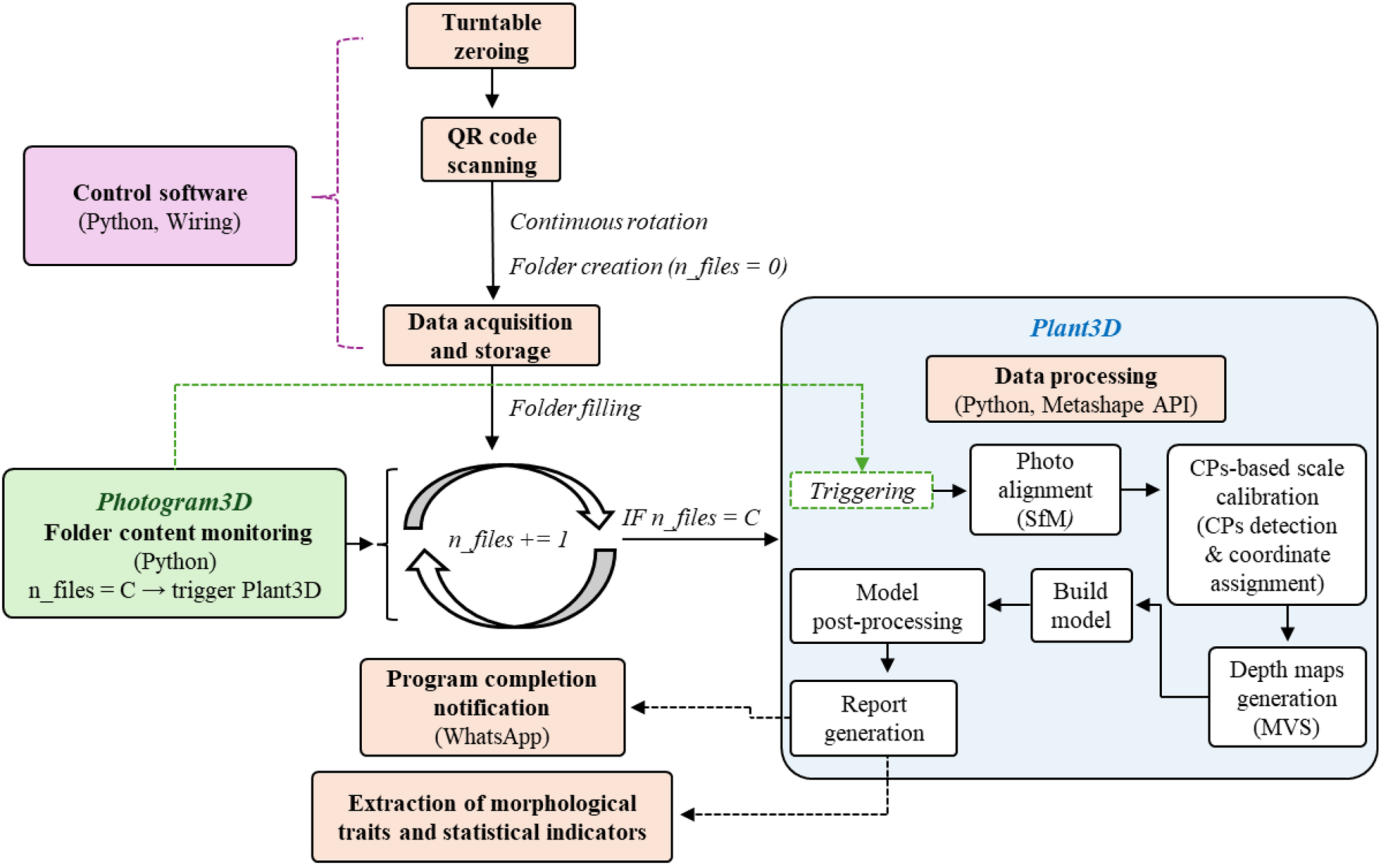
Simplified diagram of the developed photogrammetric approach, illustrating automated data acquisition, evaluation, and processing, with control points (CPs) used for scale calibration. The output consists of a report on the morphological features of the reconstructed model and the associated key statistical indicators

The acquisition and processing of data was predominantly automated through the use of Python-based scripts. Before starting photogrammetric analysis, a Photogram3D programme was executed to cheque the contents of the target folder to which the data were subsequently directed. The evaluation process was automatically started after reaching a predefined number of images in the specified directory. The following section provides a more thorough description of these steps. The image acquisition, incorporating the control of the robotic arm movement, was also facilitated by Python scripts, while the motion of the turntable was managed by a script in Wiring. The timing of camera activation, robotic arm movement, and turntable rotation speed was automated by a wrapper control script. The initial step of the data acquisition process involved automatically scanning the QR code affixed to the surface of the cuvette once the object was correctly positioned and clearly visible to the camera. The code contains the identification data, i.e., the name of the sample and its abbreviation. Based on the decoded information, a folder was created and named. The naming process incorporated a date that was automatically generated by the script. The images were then stored in the designated folder. After a scan of a single object, the folder contained a maximum of 360 images in the data set. The described process is illustrated in a simplified form in Fig. 3. The complete scanning process was completed within a maximum time frame of 8 min, and as part of the optimisation process, an attempt was made to reduce this time. To assess the reliability of the results, each object was subjected to three scanning cycles. The 3D model was generated via a custom application called Plant3D, which was developed as part of this study and features its own graphical user interface and console output. Plant3D was launched by a supervisory programme (Photogram3D) at the beginning of the analysis to perform photogrammetric processing. The application utilised the imported Metashape module (Agisoft Metashape API) for fully automatic spatial reconstruction [49]. The initial step of data processing involved alignment of the images through the structure-from-motion (SfM) algorithm, with adaptive fitting enabled and automatic filtration of the stationary points. As part of the alignment phase, the parameters of the internal orientation were also determined. The subsequent step involved the detection of 16 circular 12-bit control points and the automatic assignment of the corresponding coordinates. This step ensures the definition of the coordinate system and scale of the digital workspace and provides information about the resulting accuracy of the spatial reconstruction. Due to the size of the samples, processing was performed in millimetres. Following the creation of a sparse point cloud consisting of the detected tie points, a predefined bounding box was automatically set, limiting the reconstruction area for further processing (scanned plant and upper part of the cuvette). This cropping was implemented to reduce computational time, as the focus of the study was not on the turntable model with a cuvette. The generation of depth maps was performed via the multi-view stereo (MVS) algorithm, which builds upon previously computed camera positions from SfM. This step was performed with the highest quality settings and medium-level filtering. Additionally, according to the developers’ recommendations for the reconstruction of thin-walled structures, custom tweaks (“ooc_surface_blow_up“, “ooc_surface_blow_off“) were utilised [50]. The selection of a designated filtering mode results in the delicate elimination of extraneous points from a dense point cloud, while ensuring the preservation of the finer details and structures of the object. The actual generation of the spatial model of the plant, represented as a mesh, was carried out through depth masks of the images. Post-processing of the generated model involved automatic partial surface smoothing (strength level 1), a combination of manual and partially automated removal of isolated patches from the surroundings, and the closure of holes, e.g., at the bottom of the plant stem. The generation of a report containing all descriptiveparameters, e.g., the qualitative andstatistical indicators, and the processing and model generation times, was initiated before the termination of the script. Upon completion of the entire evaluation process, a notification was sent. This approach resulted in substantial acceleration and streamlining of the evaluation process for many scanned objects. For the entirety of the optimisation procedures, only models without texture and colour information were generated, as the emphasis was exclusively on their morphological characteristics. Consequently, no requirement was placed on the calibration of colour hue or surface reflectance. The HP Z2 Workstation desktop computer (CPU: 13th Gen Intel Core i9, GPU: NVIDIA RTX A4000 16 GB, RAM: 32 GB) was used to process the image data and generate a 3D model of the object, due to the substantial computational demands of photogrammetric reconstruction. All data were stored and backed up on a Samsung SSD 870 QVO 8 TB, with a secondary backup stored in the OneDrive cloud. In this work, all 3D models were visualised in a shaded, artificially coloured form.

### Photogrammetric reconstruction optimisation

#### Camera calibration

Since a non-metric camera with initially unknown interior orientation parameters was used for image acquisition, particular attention was given to assessing its impact on the precision of image alignment during processing. To mitigate potential instability in camera parameters over time, the effectiveness of pre-calibration functionality available in Agisoft Metashape was evaluated. Four calibration strategies were compared. The first used fixed interior orientation parameters obtained by pre-calibration, hereafter referred to as “Fixed”. The remaining three strategies involved simultaneous calibration during image alignment, with varying degrees of prior information. The second approach used pre-calibrated parameters as starting values, but allowed adjustment during processing (“Simul.”). The third approach provided only basic physical characteristics of the camera-pixel size and focal length without performing pre-calibration (“No calib.”). The fourth strategy excluded all a priori information (“No info.”). Pre-calibration was conducted in Agisoft Metashape using 18 images of a black-and-white checkerboard pattern, generated by the software and placed at the same position as the scanned object. Importantly, the entire image frame was filled with the pattern and uniformly illuminated to ensure optimal results. The comparative evaluation was carried out on three samples for each plant species. The alignment quality was consistently set to the highest level, with no limit on the number of detected keypoints. Additionally, processing was conducted with and without the adaptive camera model fitting (ACMF) option, a feature especially recommended for use with uncalibrated cameras, wide-angle lenses, or scenarios involving changes in camera orientation during image acquisition. Coded targets placed on a turntable were used for the assessment, with eight designated as control points and eight as check points.

#### Exposition and tweak setting

As part of the optimisation of the imaging parameters, the following exposure times were chosen: 30, 40, 50, 60, and 70 milliseconds. For this and all subsequent experiments, the depth-of-field was set so that the scanned object was sharp in all images at all 6 different height levels. Scanning thin, small, and detailed objects is generally associated with the problem of producing non-compact, leaky models [50]. This problem can be solved by setting up advanced features called “tweaks“. Based on the recommendations of the developers of Agisoft Metashape software, advanced settings were applied during the “Build Model“ phase, where the model was generated from depth maps. These parameters operate by expanding or contracting the surface contours, analogously to the morphological operations of dilation and erosion known from image processing but applied within a three-dimensional context. To find the best setting for each plant species, the following values were tested and evaluated: 0.95, 0.9, 0.7, 0.5, and 0.3.

#### Object and camera position

The scanned objects displayed both morphological diversity and fine structural detail, characteristic of each plant species examined. Consequently, a series of measurements were carried out using varying object-to-camera distances to determine the optimal imaging distance between the camera and the plant for each species. The distances selected for this purpose were 12, 14, 16, and 18 centimetres. Each distance was set for all six height levels (P1-P6). The lowest distance was predicted to provide the best detail capture, whereas the highest distance was expected to facilitate the alignment of images during model generation. The lowest distance could not be further reduced, as the minimum focusing distance of the lens is approximately 10 centimetres (plus a margin), according to the technical specifications. The highest distance was determined on the basis of the limitations of the workspace, size of the objects to be scanned, and camera field-of-view. Finally, a combined configuration of 12 and 16 centimetres was also defined, with the shorter distance applied at the positions P1, P3 and P5 and the longer distance at the positions P2, P4 and P6.

#### Optimisation of computational and scanning efficiency

Processing a substantial quantity of the acquired images was both time-consuming and computationally intensive. Consequently, it was imperative to reduce the number of images while preserving the integrity of the generated 3D model. To this end, a reduction protocol was defined. The fundamental components of the protocol are delineated in Table 2. The symbol X on the label denotes the plant species. The green square indicates the images taken from a given height level (P1-P6). The marking of all positions (X_2) with a degree of reduction of -1/2 represents the fraction of the images used, specifically a situation in which every second image in each marked position was omitted. In other words, the total number of images (360) for a given object is reduced by a factor of two to 180. In particular, a similar procedure was followed in the remaining cases.

**Table 2:**
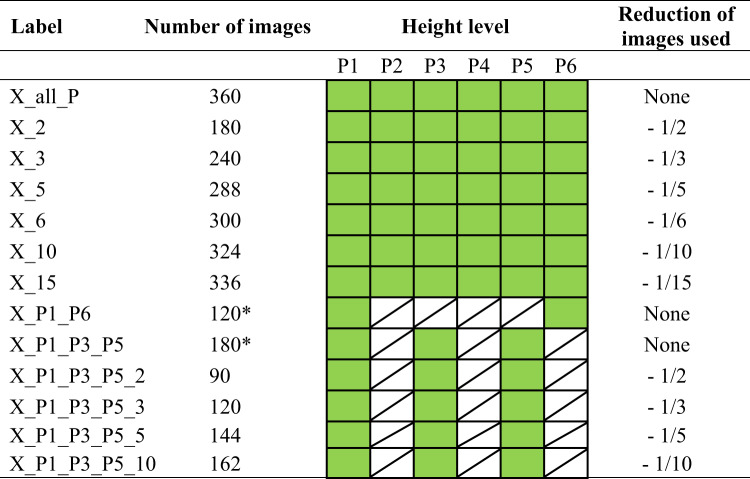
Summary of image counts aimed at reducing the scanning and processing time in the generation of 3D model

### Evaluation of the suitability of parameter settings

The suitability of the chosen settings for the parameters studied that define the photogrammetric reconstruction was evaluated on the basis of several key criteria. The first set of criteria was devoted to the qualitative characteristics of the generated mesh model, with a particular emphasis on surface integrity and volume accuracy. The second criterion involved the analysis of statistical indicators. The quality of the photo alignment was assessed using qualitative indicators, including the number of tie points (TP), the root-mean-square reprojection error in pixels (RMS RE), the maximum reprojection error in millimetres (Max RE), and the average tie point multiplicity (AVG TP M). The evaluation metrics also included differences between the known and estimated positions of the control and check points, namely the mean absolute error in millimetres (MAE), the root-mean-square error in millimetres (RMSE), and the standard deviation (SD) of the alignment error. In subsequent stages of the work, all coded markers were treated as control points. Consequently, RMSE was referred to as the control point error (CPE) and was used as an indicator of the overall spatial precision reflecting the mean discrepancy between the measured and true coordinates of the 16 circular reference control points that defined the scale of the digital workspace. The quality and detail of the generated 3D model were assessed in terms of the total number of faces and vertices. Table 3 provides a detailed description of the individual statistical indicators. The statistical analysis was also accompanied by a manual image analysis of the model and served as an important concluding criterion in the overall evaluation. This analysis focused primarily on the presence of relics or irregularities on the model surface, imperfections within the model morphology, or the presence of holes even after adjustments by tweak settings had been made.

**Table 3:** Key indicators used in photogrammetry to assess the quality of the 3D model and related processing steps

Workflow stage	Indicator*	Description
Photo alignment	TP	The key points are automatically detected and matched between the overlapping images to determine the relative orientation. A higher number usually makes alignment more accurate.
	RMS RE, RMSE	Shows the average distance (error) between the reprojected tie point and its observed position in the image. Lower values are better.
	Max RE	The largest error was observed when reprojecting the tie points across all images, corresponding to the identification of the worst-case alignment error.
	AVG TP M	The mean number of images in which each tie point is visible. Higher values indicate greater redundancy and, therefore, more robust alignment.
Coordinate system accuracy	CPE	The error between the known and calculated coordinates of the control points indicates an error in the georeferencing of the model. This is indicative of the absolute accuracy of the model in real-world coordinates.
Model reconstruction quality	Faces	The total number of polygonal faces (typically triangles) in the 3D mesh corresponds to a specific level of complexity.
	Vertices	The individual points in 3D space that define the shape of the mesh by forming the corners of faces (typically triangles or polygons) are known as vertices. An increased number of vertices is indicative of greater geometric complexity and detail.
	Surface	The term used to denote the level of detail, resolution, and noise level of the reconstructed 3D surface. A high-quality surface is characterised by its intricate detail and the absence of artefacts.
	Volume	The purpose of this indicator is to provide a quantitative measure of the precision of the volumetric calculations derived from the model. The accuracy of the results depends not only on the correct geometry and scale, but also on the advanced settings of the 3D model generation procedure.

* All parameters were calculated using Agisoft Metashape and were part of the automatically generated processing report.

The reliability and usability of the photogrammetric system were indirectly benchmarked against previously tested equipment, including the POP 3 3D scanner [48]. In the referenced study, this low-cost scanner was quantitatively compared with several more precise and expensive commercial 3D scanners and was shown to be the most suitable for reconstructing small and detailed plant structures. In the present work, the POP 3 scanner was replaced by a photogrammetric approach using an RGB camera, representing a further methodological improvement. Image analysis of the generated models confirmed that the photogrammetric system is even more suitable than the low-cost scanner, and thus, by extension, also more suitable than the high-end devices included in the earlier comparison. This step was essential for evaluating the potential to expand the usability of the multifunctional equipment under development, with the aim of generating high-quality 3D plant models and enabling non-destructive studies of their morphological traits.

## Results

In the following chapter, the results are clearly presented, visualised, and interpreted primarily through graphs and illustrative figures. This chapter offers an overview of the most suitable parameter configurations for optimised photogrammetric analysis, with particular emphasis on the qualitative attributes of the generated 3D model of the scanned object.

### Camera calibration

**Fig. 4:**
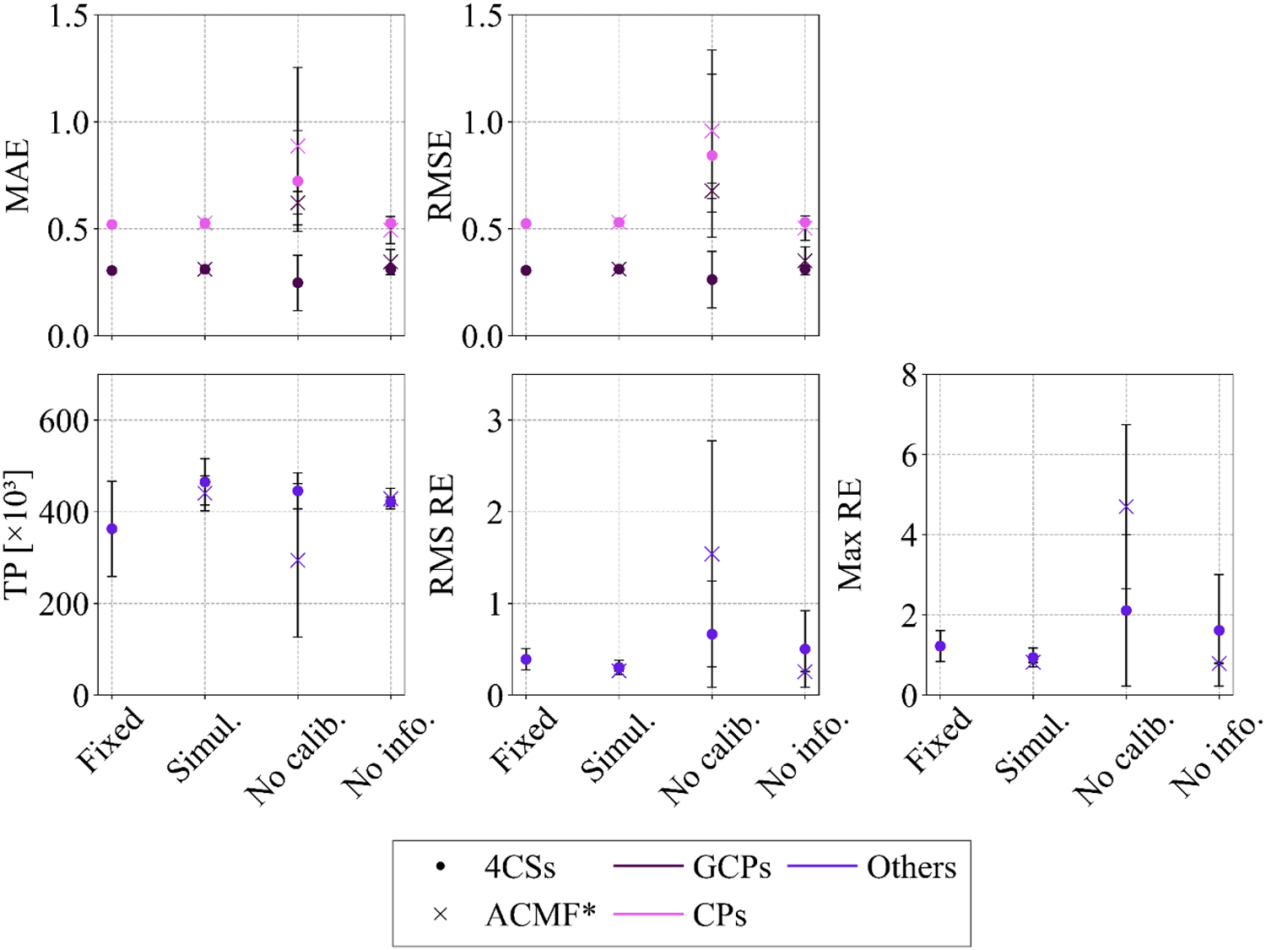
Comparison of four camera calibration strategies for all plant species, assessed by accuracy (MAE, RMSE), alignment quality (TP), and reprojection errors (RMS RE, Max RE). Results with adaptive camera model fitting (ACMF) are included. * Processing with adaptive camera model fitting using only focal length and pixel size (“No calib.”) led to partial alignment failure. This configuration was therefore excluded from the results

The results confirmed the anticipated influence of unknown and potentially unstable interior orientation parameters on the quality of spatial reconstruction. The highest accuracy measured by residuals at the control and check points, was obtained via simultaneous calibration accompanied by information about the interior parameters derived from pre-calibration (see Fig. 4). However, the most consistent reconstruction performance across repeated trials was observed with the simultaneous calibration approach using pre-calibrated values as initial estimates, underscoring the robustness of this second strategy. Although the specific effects of the adaptive camera model fitting (ACMF) option are not discussed in depth, the tests indicated a pronounced positive impact when sufficient prior information was available (“Simul.”) or when no prior information was provided (“No info.”). In contrast, the strategy using only physical camera parameters, focal length, and pixel size (“No calib.”) resulted in notably reduced accuracy, and in the case of *L. sativa*, it led to partial image alignment failure. Despite the variability between scenarios, adaptive fitting generally improved both the accuracy and geometric consistency of the reconstructions. As such, its use was considered advantageous and was applied in all subsequent processing steps. Ultimately, the simultaneous calibration approach incorporating pre-calibrated parameters of interior orientation and ACMF was selected for continued use in the photogrammetric workflow.

### Exposure time and tweak setting

**Fig. 5:**
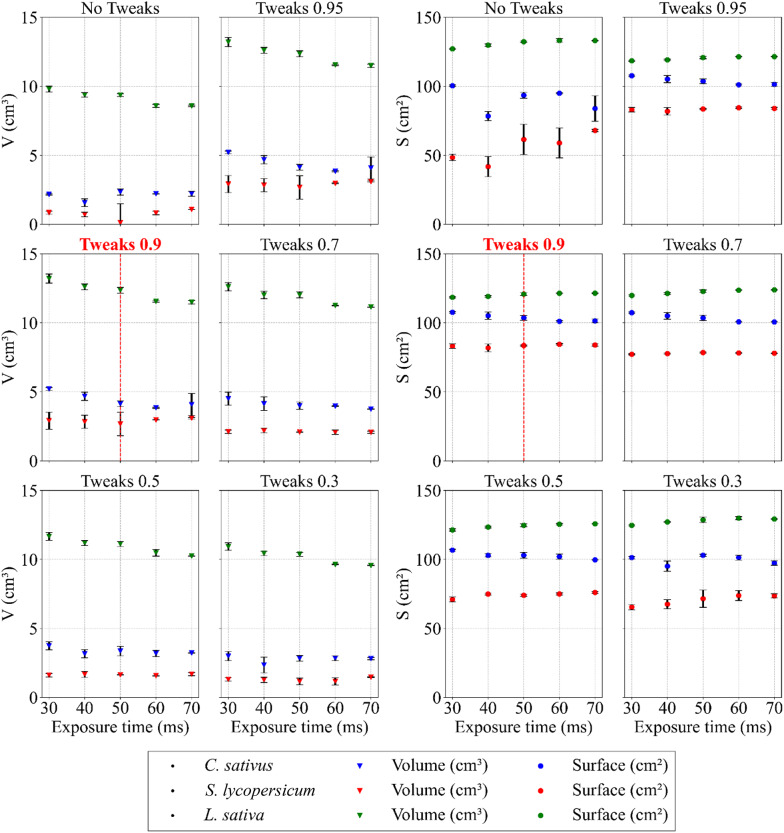
Combinations of exposure times, tweak settings, and plant species in relation to the qualitative features of the 3D models (volume and surface). The selected parameters are coloured red. Data are shown as mean ± SD (*n* = 9 plants per species and setting)

The average values of qualitative features, namely volume and surface area, were obtained for each plant species in the full spectrum of predefined tweak settings and exposure times (see Fig. 5). These results illustrate the variability of 3D model characteristics under different parameter combinations and allow the identification of optimal settings. To enhance clarity, the volume and surface values are presented separately as a result of their different order levels.

As tweaks are not enabled by default in Metashape Agisoft, their effect on reconstruction quality was systematically tested. When scanning thin structures, holes and imperfections frequently occurred in the generated models. The data confirmed the positive impact of virtually any tweak setting on model quality. An increase in tweak values was generally associated with improved reconstruction accuracy (see Fig. 5). In cases where no tweaks were applied (“No Tweaks”), the negative impact was most pronounced on the surfaces of the *C. sativus* and *S. lycopersicum* plant models. In contrast, volume values remained relatively unchanged with this configuration, while the surface and volume of *L. sativa* models were found to be relatively resilient. Across predefined adjustment values, the resulting trends were similar for all plant species.

An increase in tweak values led to a noticeable reduction in surface holes in the generated models. This improvement was largely attributed to applied operations, such as dilation and erosion, which compensated for missing data by filling structural gaps. A slight thickening of some plant structures, particularly the leaves, was observed as a side effect of this process, leading to a modest overestimation of the total volume. However, the advantages gained through this optimisation step outweighed the minor limitations. In the case of *L. sativa*, a slightly decreasing trend in volume was observed with increasing exposure time on all adjustments, possibly related to lighting and lower image quality. Exposure time within the tested range was not a significant factor that influenced the features of the model, and for subsequent optimisation steps, the median value of 50 milliseconds was selected, as it avoided both underexposure and overexposure. The ambiguous impact of the tweak settings on model quality precluded the selection of a single most suitable option at this stage.

**Fig. 6:**
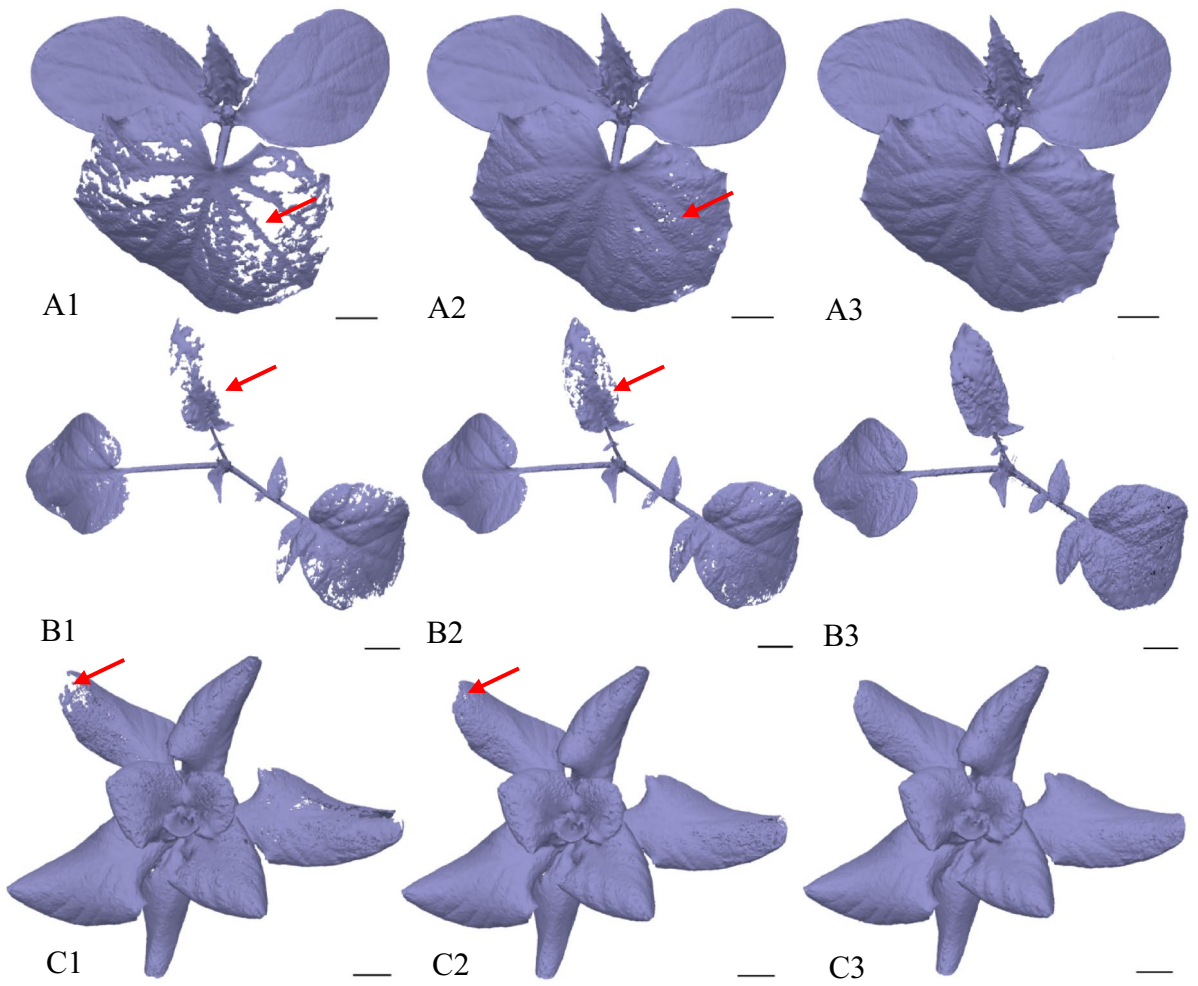
Illustrative series of additional images showing the effect of different tweak settings on model quality and integrity. Each image includes a black 1-cm scale bar in the lower right corner. Numerical codes correspond to tweak levels: 1 corresponds to “No Tweaks“, 2 to 0.3, and 3 to 0.9. Species are labelled as follows: A denotes *C. sativus*, B denotes *S. lycopersicum*, and C denotes *L. sativa*. The red arrows highlight the regions where holes and imperfections occurred

Detailed analysis of the generated model images served as an important complementary assessment of the correctness of both the tweak settings and the exposure time, and this image-based assessment confirmed the validity of the preliminary parameter configuration (see Fig. 6). A tweak value of 0.9 was ultimately selected as the most effective setting, as at this value there were no holes in the models, which makes them complete and compact. Retrospective analysis of data trends (see Fig. 5) further showed that the presence of holes had a negligible effect on the total volume and surface area. In summary, the segment of the model comprising holes was found to be inconsequential in relation to the total mass of the model, underscoring the robustness and relative reliability of the introduced photogrammetric approach with respect to model quality, independent of plant species.

### Object and camera position

**Fig. 7:**
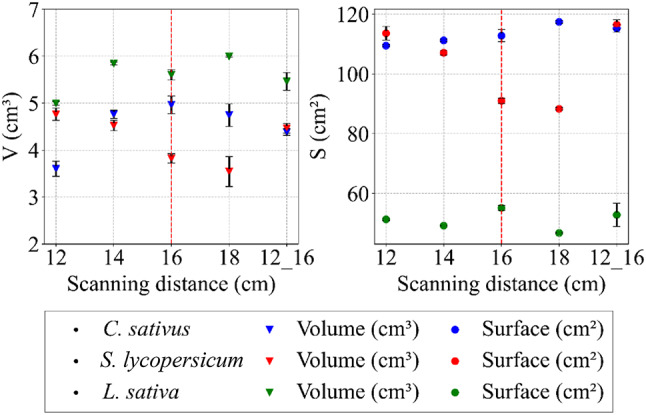
Quality indicators of models in all plant species as a function of the distance between camera and object. The red dashed line indicates the optimised distance selected for further processing. Data are shown as mean ± SD (*n* = 9 plants per species and setting)

**Fig. 8:**
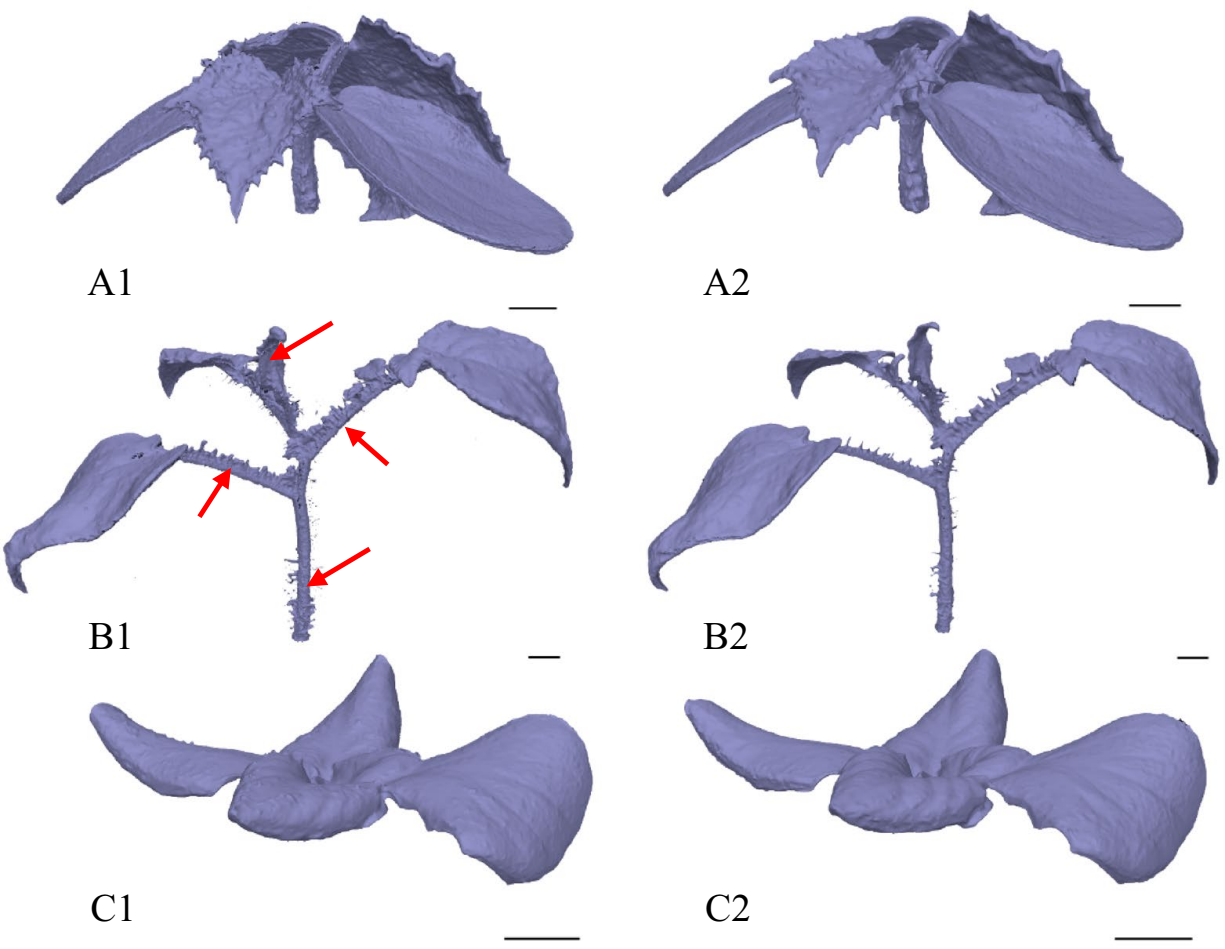
Illustrative series of auxiliary images showing the effects of camera–object distance on model quality and integrity. Each image includes a black 1-cm scale bar in the lower right quadrant. Numerical codes denote specific distances: 1 corresponds to the shortest distance of 12 cm (with comparable features also at 14 cm and a mixed 12/16 cm setting), and 2 corresponds to 16 cm (with similar features also at 18 cm). Species are labelled as follows: A – denotes *C. sativus*, B – denotes *S. lycopersicum*, and C – denotes *L. sativa*. The red arrows highlight areas where artefacts and other imperfections were observed

Analysis of surface data and related trends for the *C. sativus* and *L. sativa* models indicated that the distance between objects and the camera did not significantly affect the results (see Fig. 7). In contrast, for *S. lycopersicum*, the lower distances (12, 14 and the combination of 12 and 16 cm) tended to overestimate surface values. At very close distances, even small trichomes on the stem surface were detected; however, their reconstruction produced unrealistic artefacts, which slightly increased the total surface area. With respect to volume, no specific trend was observed for any species, indicating that object-to-camera distance did not exert a substantial influence on this parameter. Supplementary image analysis (see Fig. 8) highlighted imperfections caused by suboptimal distances and was used to support the determination of an optimal scanning distance.

**Fig. 9:**
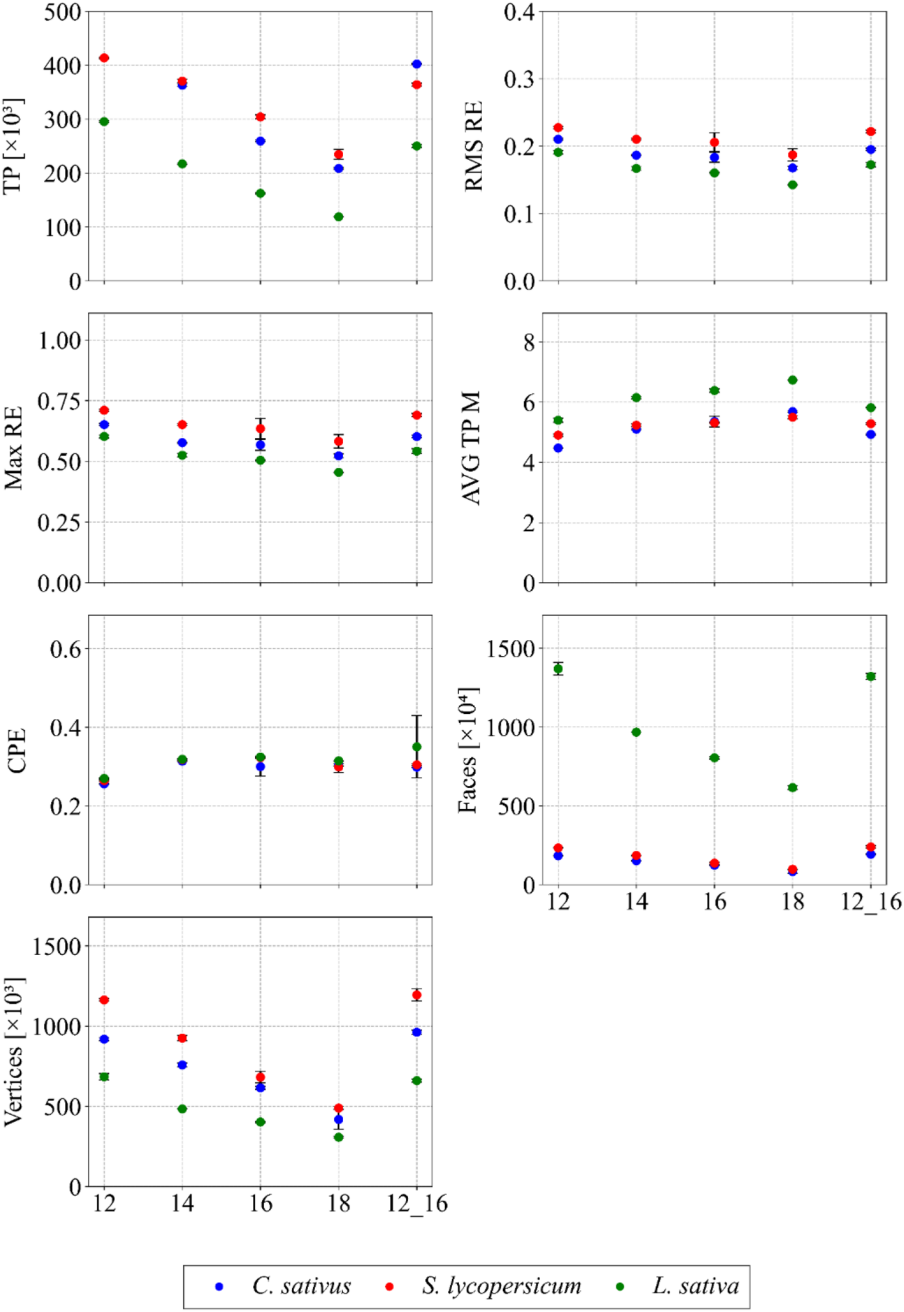
Trends in key statistical indicators in relation to the distance between camera and object in all plant species examined. Data are presented as mean ± SD (*n* = 9 plants per species and setting)

The RMS RE, Max RE, AVG TP and CPE values were consistent between species and did not show discernible changes with varying object-to-camera distance (see Fig. 9). An additional statistical indicator confirmed the limited influence of distance on model quality, and other parameters, including TP, faces, and vertices, supported these findings. Scanning at distances closer than 16 cm generated artefacts, leading to a slight overestimation of these quality metrics, while increasing the distance to 18 cm reduced face and vertex counts due to lower detail capture. The statistical indicators thus provided a valuable complement to the assessment of this optimisation step. Based on a holistic evaluation,the optimum distance for this configuration was determined to be 16 cm, however, for larger plants, this step would need to be repeated, as the optimal scanning distance is likely to increase.

### Optimisation of computational and scanning efficiency

**Fig. 10:**
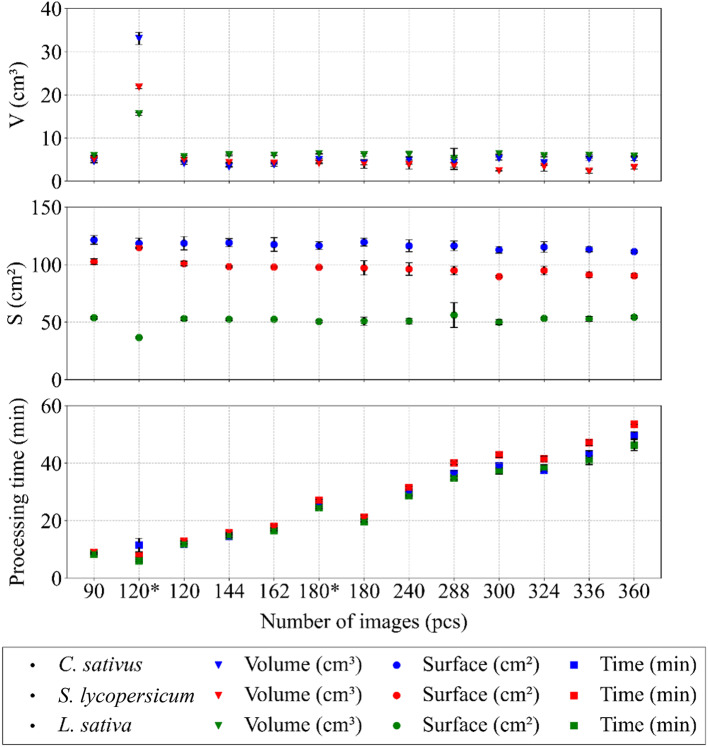
Representation of the robustness of the photogrammetric approach in relation to the number of frames and the processing time required to generate a 3D model, evaluated with respect to model quality (volume and surface area). Data are presented as mean ± SD (*n* = 9 plants per species and setting)

**Fig. 11:**
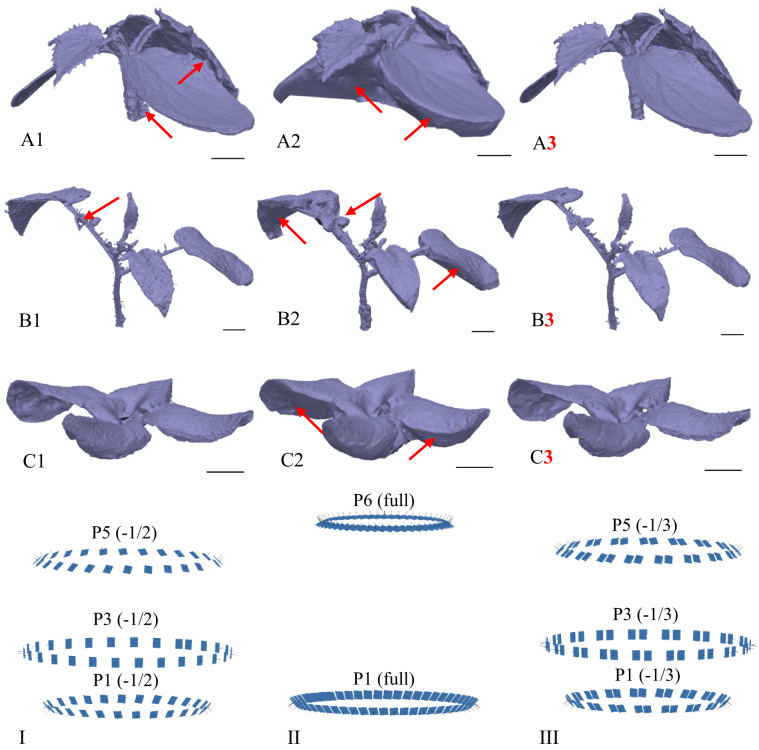
The diagram illustrates the influence of frame number and image configuration on model quality. Each image includes a black 1-cm scale bar in the lower right quadrant. For demonstration, models generated from the most relevant datasets in the optimisation process were selected: 90 (1), 120 (2), and 120 (3) frames. Red arrows indicate imperfections such as holes or redundant artefacts arising either from an insufficient number of frames (1) or an unsuitable configuration (2). The red bold number highlights the model generated with the lowest frame count that still maintained acceptable quality. Species are labelled as follows: A – denotes *C. sativus*, B – denotes *S. lycopersicum*, and C – denotes *L. sativa*. The lower part of the diagram depicts the spatial image configurations of the datasets. Roman numerals distinguish the cases: I – frames of P1, P3 and P5 with a 50% reduction per level; II – frames from P1 and P6 with 60 images per level; III – frames from P1, P3 and P5 with a one-third reduction per level

The subsequent trend in the processed data demonstrated a positive correlation between the number of frames and the time required to process the data and generate a compact 3D model (see Fig. 10). In other words, an increase in the number of frames was consistently associated with a longer processing time. The indicators of surface and volume were largely unaffected by the decrease in the number of images, which confirms the robustness of the photogrammetric approach and the reliability of the evaluation process. A substantial trend fluctuation was observed in the case of “120*“. This irregularity can be attributed to the failure to establish sufficient tie points between the two image sets caused by considerable differences in camera positions and viewing angles. Consequently, the generation of a model of sufficient quality was rendered impossible and the resulting models contained the most discernible imperfections throughout the study (see Figs. 11 – A2, B2, C2). In the case of “180*“, an increase in processing time was observed that did not correspond to the general trend. However, no such increase was observed in the values of the qualitative features (surface, volume), indicating that the overestimation was due to software data processing rather than actual changes in model quality.

The outcome of this optimisation step was the identification of the minimum number of frames and image configuration required to produce a high-quality 3D model. From this perspective, the second-lowest frame count tested was selected, specifically 120 images, with 40 images captured at each of the height levels P1, P3 and P5. It was demonstrated that at this frame count and configuration, the resulting model quality was practically equivalent to that of the model generated using the full set of 360 original frames from all height levels. The lowest number of images tested, 90 (P1, P3, P6 with 30 images at each level), was deemed insufficient due to imperfections and redundant artefacts in the resulting models, particularly for *C. sativus* and *S. lycopersicum* (see Fig. 11 – A1, B1), while in the case of *L. sativa* the quality of the models was relatively comparable to those generated from 120 images (see Fig. 11 – C1, C3). A substantial discrepancy was observed between 120 and 120. In both cases, the initial datasets contained 120 images, but differed in image configuration and frame reduction (Table 2). It can be concluded that reducing the number of frames while preserving a greater number of positions was a more appropriate optimisation procedure than reducing the number of positions while maintaining the full number of frames at a given position, a conclusion based on the resulting model quality and fully consistent with photogrammetric principles.

**Fig. 12:**
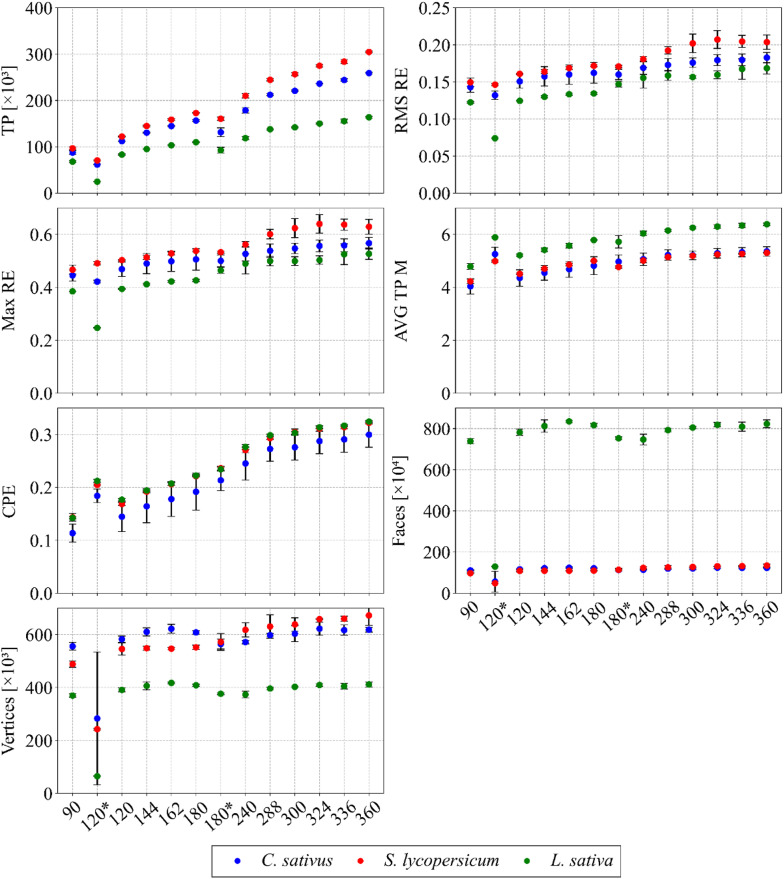
Predominantly increasing trends in selected quality indicators with respect to the number of processed images in all plant species. Data are presented as mean ± SD (*n* = 9 plants per species and setting)

The resulting values of crucial statistical indicators that describe the quality of the generated 3D models corroborated the preceding claim, confirming that 120 images represent the minimum quantity required to generate a plant model of sufficient quality. A similar trend was observed across all indicators, namely an increase in their values with an increasing number of images for all plant species (see Fig. 12). As the number of frames increased, the software detected a greater number of features, which resulted in an increasing trend in the data for TP, faces, and vertices. The inadequate degree of image linkage (observed in the case of “120*“) was systematically reflected in all the analysed indicators. A decrease in the number of TP was also recorded in the case of “180*“, which may have been influenced by the reduced number of frames, although the other indicators in this case did not differ significantly.

The most significant outcome of this optimisation stage was the substantial reduction in processing time and computational complexity associated with image processing and model generation, which reached approximately 75% across all plant species. To further contextualise this finding, it should be noted that when the lowest tested number of images (90) was used, the time and energy savings would have reached about 82%, but at the cost of the adverse effects already described (holes, artefacts). Another, less evident but noteworthy improvement concerned the optimisation of the minimum number of frames used. By theoretically eliminating the need to scan up to 360 images per plant (a process lasting about 8 min), the scan time could be reduced by one third, to approximately 2.7 min, thereby enhancing the overall efficiency of photogrammetric reconstruction. This optimisation step substantially extended the applicability of the multifunctional robotic scanning apparatus [48].

### Assessment of plant morphology

The examined plant species exhibited substantial variation in their morphological characteristics. The size and number of thin parts of the objects posed a challenge for photogrammetric reconstruction. The morphology of *C. sativus* is characterised by the presence of a relatively thick stem and twotypes ofleaves: cotyledon leaves with smooth edges and true lobed leaves with serrated edges. This specific constitution was conducive to the scanning process, resulting in complete models free of unwanted artefacts. The morphology of *S. lycopersicum* is characterised by the following traits: the presence of oval-shaped leaves with smooth edges, thin petioles, and a slender stem covered with thin trichomes that were imperfectly reconstructed. In the majority cases, the resulting model was composed predominantly of swollen redundant trichome artefacts, which slightly overestimated the values of the resulting qualitative indicators. The morphological traits of *L. sativa* are characterised by the presence of a dense rosette of overlapping and twisted drop-shaped leaves with smooth edges. The primary issue encountered during the scanning process was that the dense rosette obscured the interior from the camera’s view, preventing effective penetration and thorough scanning. In this case, the volume and surface area were also likely to be slightly overestimated.

**Fig. 13:**
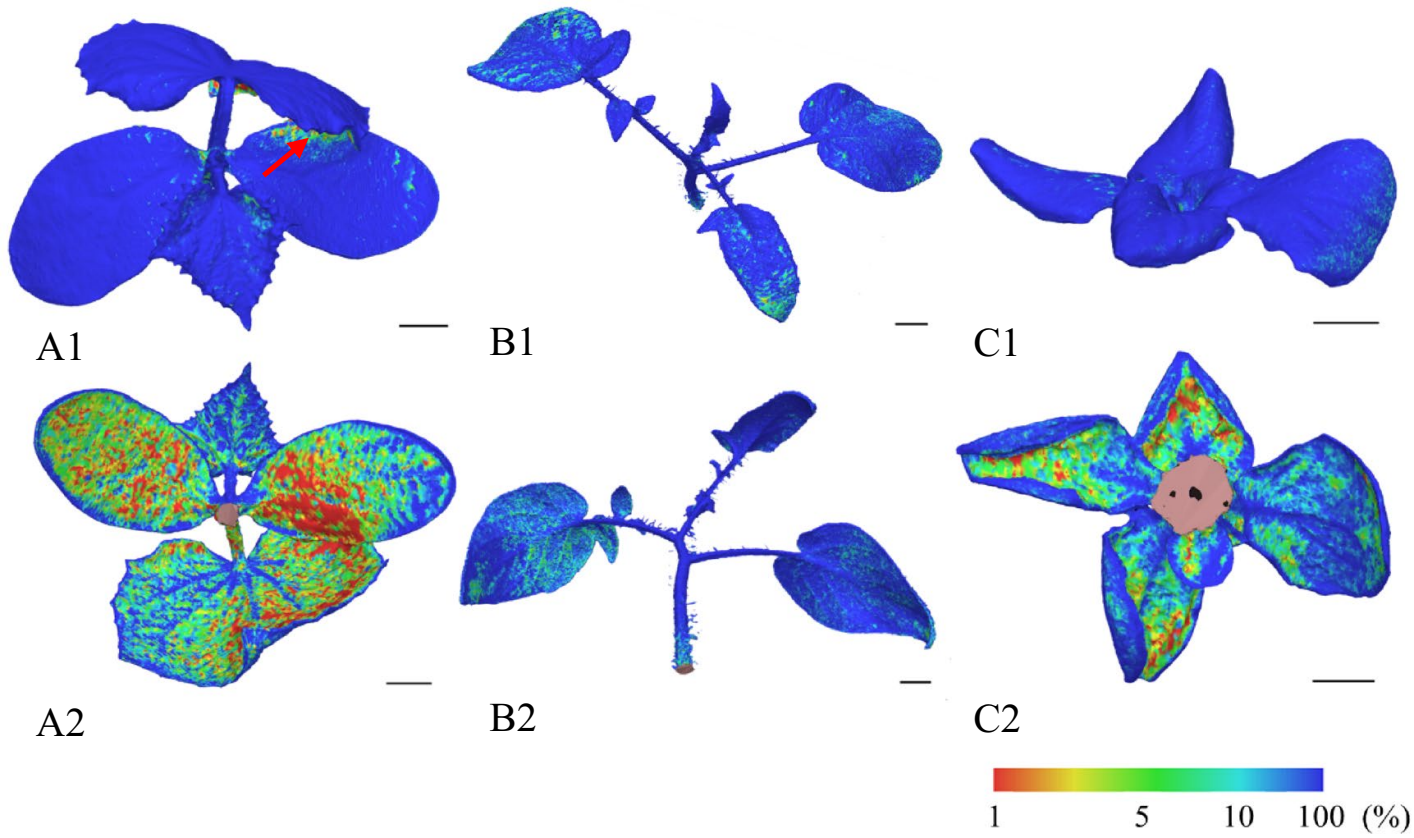
The visualisation shows model confidence levels for all plant species. Each image contains a black 1-cm scale bar in the lower right quadrant. Confidence is expressed on a normalised scale from 1 to 100%. Numeral 1 denotes the upper side of the plant and numeral 2 the lower side. Species are labelled as follows: A denotes *C. sativus*, B denotes *S. lycopersicum*, and C denotes *L. sativa*. The red arrow marks an area of low certainty caused by overlapping leaves and limited camera access

The impact of the morphological diversity of the plants on model quality was evaluated by analysing the confidence level of point reconstruction from the original image data. This indicator expresses the degree of precision and reliability in reconstructing the meshes, visualised on a standardised scale from 1 to 100, where higher values indicate greater reliability (see Fig. 13). The image data were acquired with the photogrammetric system set to the optimal parameters identified above. For *C. sativus*, the areas on the underside of the cotyledon and the true leaves exhibited relatively low confidence levels (1–20%), which may have been caused by the short stem (2.5 cm) and drooping leaves that reduced the handling space in the lower part of the plant and limited camera accessibility; overlapping leaves also proved more difficult to scan (see Fig. 13 – A1, red arrow). In contrast, *S. lycopersicum*, with its longer stem (5.2 cm) and leaf petioles, provided a more spacious and accessible area for the camera, facilitating scanning and the acquisition of higher-quality data. For *L. sativa*, the absence of a stem and the curled, drooping leaves similarly reduced camera accessibility, which was reflected in the colour map of the model as a manifest decline in confidence levels in these areas. Despite these localised limitations, most of the mesh vertices in all species exhibited values approaching 100%, confirming the robustness of the reconstruction process. This finding demonstrates that, while morphological differences create species-specific challenges in data acquisition, the overall photogrammetric approach remains reliable across diverse plant architectures. Considering the modest financial investment in the upgraded low-cost scanning apparatus, the resulting models proved sufficiently detailed for downstream analyses, highlighting the system’s cost-effectiveness. The apparatus, with its proven reliability, thus offers a practical solution for a range of applications, from basic morphological studies to more advanced phenotypic assessments.

**Fig. 14:**
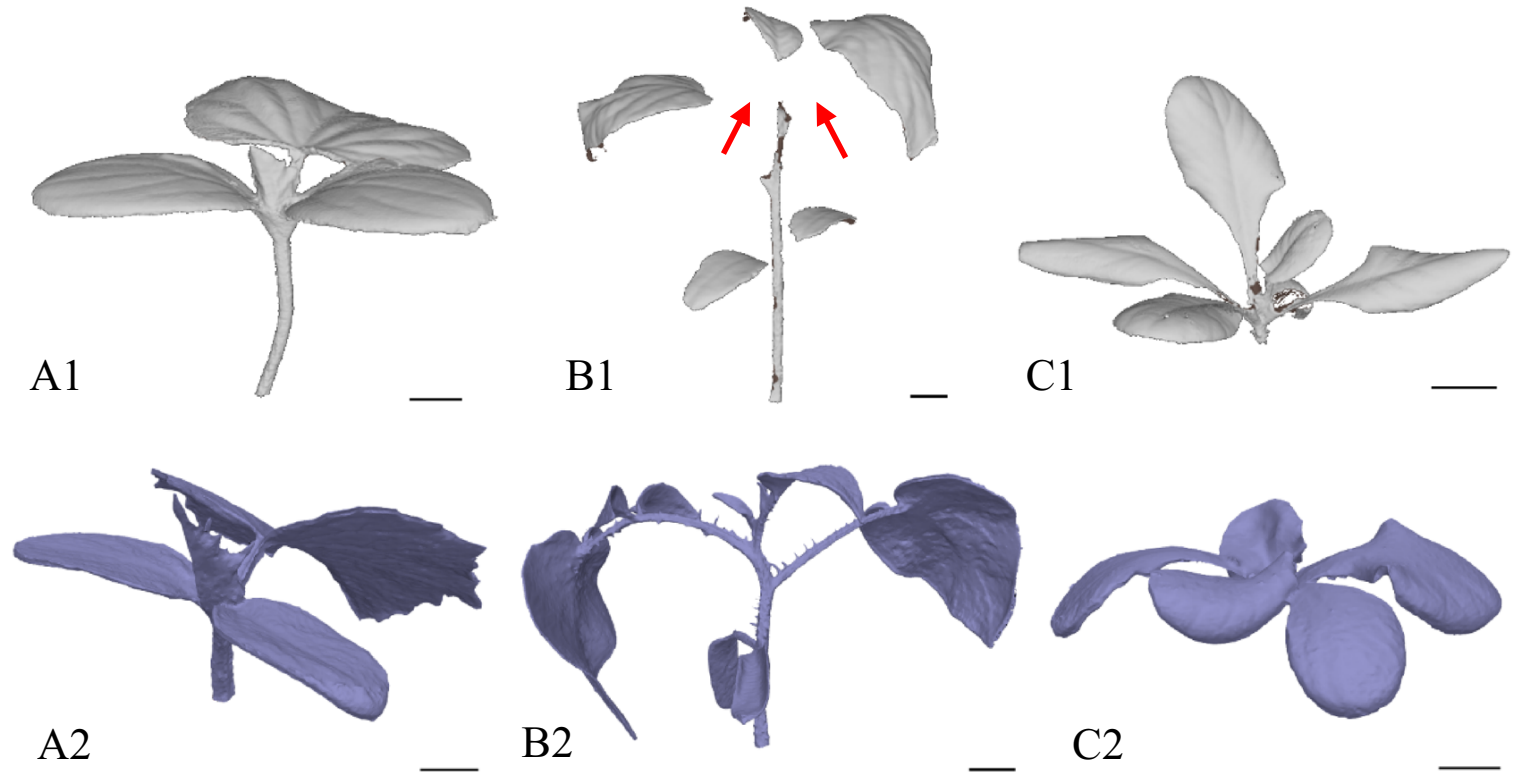
Illustrative comparison of models generated by two reconstruction approaches. Label 1 indicates grey models obtained using the 3D scanning method (POP 3 scanner with RevoScan 5 software), whereas label 2 denotes purple models produced with the optimised photogrammetric workflow. Each image includes a black 1-cm scale bar in the lower right quadrant. Species are labelled as follows: A – denotes *C. sativus*, B – denotes *S. lycopersicum*, and C – denotes *L. sativa*. The red arrows highlight areas of imperfect reconstruction in thin structures of *S. lycopersicum*, particularly in slender leaf petioles

Figure 14 shows a comparison of the resulting plant models used to assess the usability of the optimised photogrammetric system. In contrast to the previously tested POP 3 3D scanner, image analysis demonstrated that the photogrammetric system could generate complete and high-quality models. Data from the POP 3 3D scanner were evaluated using RevoScan 5 software, which did not support scripting and was therefore unsuitable for the automated workflow implemented in this study. The models produced by the 3D scanner were often incomplete, particularly due to difficulties in capturing the underside of plant leaves, making it impossible to generate reliable values for volume and surface area or to determine descriptive quantitative parameters. For *S. lycopersicum*, thin leaf petioles posed a particular challenge (see Figure 14 – B1). By contrast, the photogrammetric approach provided greater detail and completeness. Importantly, this comparison served as an indirect ground-truth benchmark. It compared an RGB-camera-based pipeline with a higher-cost commercial 3D scanner. The photogrammetric workflow matched or exceeded the scanner in completeness and metric usability, and it remained compatible with automated processing. Overall, the combination of an RGB camera and evaluation software based on the Metashape API proved to be a robust and user-friendly solution, offering significantly greater control over reconstruction algorithms than the POP 3/RevoScan setup and thus providing a more adaptable and customisable option.

## Discussion

### System configuration and calibration

Non-destructive methods of studying plant morphological features represent a contemporary biotechnological approach whose reliability and accuracy depend on the appropriate configuration of the optical system. As highlighted in earlier studies, the type of optical equipment, along with its accuracy and cost, plays a decisive role [51, 52]. Consistent with previous studies, we found that accurate reconstruction requires careful calibration of both interior and exterior camera parameters. While this is typically achieved with checkerboards or circular coded markers, in our case calibration was reinforced by 12-bit control points, which simultaneously provided ground-truthing for the calibration process. Our findings point to the conclusion that the integration of simultaneous calibration with pre-calibrated parameters and adaptive camera model fitting constitutes a particularly robust strategy for plant modelling. This supports earlier studies that highlight the importance of accurate calibration [47, 53, 54, 55, 56].

The geometry of spatial orientation of the imaging system is crucial for acquiring data from multiple positions and angles, enabling the generation of complete and high-quality 3D plant models. A key distinction of our apparatus from existing plant-scanning systems is the use of a programmable robotic arm, which enables highly flexible and precise positioning of the camera within the workspace and simplifies the definition of complex spatial arrangements. Robotic six-axis arms have been reported in competing systems and shown to deliver acceptable accuracy and reliability in 3D plant reconstruction [57]. However, the ability to achieve comprehensive spatial coverage remains a key advantage of our system and confirms that viewpoint distribution can be as critical as image quantity for model fidelity. In our case, a configuration with fewer images but distributed across more positions proved more effective than a configuration with fewer images and fewer positions. The optimal setup consisted of three height levels (P1, P3, and P5), each with 40 images taken at 10° spacing. This arrangement produced complete and reliable models. The broader implication is that distribution of viewpoints produces more accurate reconstructions, a principle also shown in archaeological photogrammetry using Agisoft Metashapes [58]. By contrast, many commercial devices capture only the upper part of the object. The underside of leaves is often particularly problematic, and incomplete reconstructions are common. Such models prevent reliable estimation of surface area, volume, and other key morphological descriptors, whereas in our study this limitation was effectively overcome. Most of these systems operate from only two positions using stereo-photogrammetry [26, 55, 59, 60, 61], and only a limited number can record partial views of the underside [17, 62]. General recommendations emphasise the need for uniform coverage and sufficient overlap, with inter-level angles ideally below 45° [63]. Remarkably, our apparatus produced high-quality models even with relatively low overlap. This was achieved by the spherical arrangement of the images, which provided almost 360° coverage of the plant surface. Comparable solutions, such as tiltable brackets with linear sliders, improved reconstructions in *S. lycopersicum*, but lack the versatility of our robotic-arm system [64]. These comparisons underline the novelty of our low-cost approach. It ensures comprehensive coverage and preserves authentic plant dimensions more reliably than conventional systems.

### Lighting and exposure conditions

The quality of 3D reconstruction is strongly influenced by lighting conditions, as most reconstruction methods, whether geometric or machine learning-based, rely on pixel intensity, texture, shading, and colour consistency [65]. Appropriate lighting, homogenisation, and optimisation of exposure time can markedly improve the quality of image data used for reconstruction [66]. In our system, a diffuse photographic panel was employed to homogenise illumination, though at the cost of reducing light intensity. This was considered when setting the exposure time. The side LED panels were left unhomogenised to improve the visibility of QR codes and ensure correct recognition. An exposure time of 50 milliseconds proved optimal, as confirmed by model image analysis and statistical indicators. The system also showed robustness to variation in exposure time, with model quality largely unaffected, consistent with findings from other studies [53, 55, 67]. Even illumination from all directions is generally recommended to minimise shadows, overexposure, or underexposure [68]. Minor imperfections caused by such effects were observed here, but they remained negligible. More intensive post-processing might further mitigate these artefacts, although this was not deemed necessary for reliable reconstructions. Reflective plant surfaces posed an additional challenge, particularly in *L. sativa*. Previous work has shown that surface reflectance complicates photogrammetric reconstruction [69]. Potential solutions include adjusting exposure or applying matting agents, although caution is required to avoid negative impacts on plant growth in longitudinal studies [70, 71]. In general, close-range photogrammetry under controlled conditions reduces the variability in light compared to outdoor approaches. Nevertheless, transferring the system from laboratory to field applications would require methodological adaptations to minimise the influence of fluctuating light conditions on reconstruction accuracy. The results of this study demonstrated that our system is relatively robust to differences in exposure time, which supports its potential applicability in field environments where lighting is less predictable. Ensuring robustness against such variability is critical for reproducibility and for extending the applicability of low-cost photogrammetric plant phenotyping systems beyond laboratory settings.

### Tweak parameter for thin-structure reconstruction

Close-range photogrammetric reconstruction of small and structurally complex plant organs presents several challenges. Thin leaves, fine stems, petioles and trichomes frequently caused holes, artefacts, and other imperfections in the generated models. These issues arose from the inability of the software to correctly determine the orientation of very thin surfaces. The uncertainty of whether a point belonged to the upper or lower surface of a leaf often resulted in its exclusion from the reconstruction or its incorrect assignment, producing local errors in the final model [61, 72]. The introduction of tweak parameters proved to be an effective solution. Across all species, a value of 0.9 was found to be the most suitable. In this setting, the holes and imperfections were successfully filled, while the surface area and volume showed only a slight overestimation that remained negligible for practical purposes (see Figs. 5 and 6). This modification of the reconstruction process therefore improved the completeness and consistency of the models. Importantly, it added further value to our system by enhancing the reconstruction of thin structures without requiring more complex or costly interventions. Alternative approaches have been proposed to address these challenges. A widely used technique is multi-focus stacking, particularly in micro-photogrammetry of small and detailed objects with fine structures, such as insects and flowers [73, 74]. This method overcomes depth-of-fieldlimitations by merging multiple images taken at different focal planes into a single composite with improved sharpness across the object. Although effective, it requires a substantially larger number of images, which increases both the acquisition time and the computational demands. These constraints limit its suitability for high-throughput plant phenotyping. By contrast, the tweak-based solution integrated into our workflow achieved comparable improvements in model completeness without these disadvantages. Therefore, it represents a pragmatic and efficient alternative, reinforcing the applicability of our low-cost photogrammetric system to reconstruct thin plant structures and ensuring reliable phenotypic measurements.

### Scanning distance and processing time

Another important factor influencing photogrammetric reconstruction is the distance between the object and the camera, as it determines image resolution, depth-of-field, and the precision of feature reconstruction. Under controlled laboratory conditions, where stability and sufficient camera resolution are ensured, the usable range of distances can be relatively broad. This observation is consistent with our findings. In the range between 14 and 16 cm, the resulting models showed comparable quality, with only the extreme values producing adverse effects due to loss of focus. Based on both image analysis and statistical indicators, 16 cm was identified as the optimal distance. A notable benefit of the photogrammetric system is its ability to extend the scanning distance up to a maximum of 30–40 cm [48], which is essential for reconstructing larger plants. Other systems that employ longer distances during scanning often report reduced model quality [56]. Our apparatus therefore offers a more adaptable solution. For plants exceeding the maximum adjustable height of the current design, modifications would be required. A related approach using two static cameras and a turntable has also produced models of comparable quality, and its use for root system reconstruction illustrates potential directions for future enhancements of our apparatus [75]. Similar solutions have been described for tall *Zea mays* plants, where individuals are scanned on rotating tables combined with vertically adjustable platforms [28, 29]. Integrating similar design features into our system, together with precise calibration of robotic arm positioning, turntable speed, and scanning frequency, could further improve its robustness and extend its applicability to larger plant species.

A major advantage of the developed apparatus is its ability to precisely position the camera and thereby generate geometrically unique image arrangements. This flexibility also allows straightforward adjustment of the total number of images and thus of scanning time. Conventional approaches typically capture images from only two positions [3, 56], while in our case six height levels were initially used. Optimisation reduced the original 360 images to 120 (40 images at each of P1, P3 and P5) without any loss of model quality. Comparison with a similar system underscores the clear advantage of our apparatus. Reconstructions of *S. lycopersicum* plants based on five camera positions produced models of inferior quality, as indicated by fewer tie points (20,000–150,000) and higher RMS RE values (0.2–0.8) [76]. By contrast, our models contained 120,000–300,000 tie points and achieved RMS RE values between 0.1 and 0.22. These results confirm that our system provides more reliable reconstructions and superior model fidelity compared with existing approaches. An additional benefit of reducing the number of images was a substantial reduction in scanning time, from 8 min to only 2.7 min per plant. This was accompanied by a marked decrease in processing time for image data and model generation, with overall savings of approximately 75%. Rossi et al. introduced a low-cost system with a rotating plate, but their reconstructions often required several hours and the fixed acquisition geometry limited adaptability [30]. Wu et al. presented a portable device that achieved faster acquisition, although it relied on predefined circular paths and was associated with higher costs [29]. Compared with these approaches, our system retains the advantage of low-cost while reducing both scanning and processing time through automation and optimised acquisition. It further offers flexible image trajectories, adapts more effectively to complex plant architectures, and improves reconstruction of thin structures via optimised parameter settings. Importantly, these efficiency gains were achieved without compromising quality. The system consistently produced high-quality 3D models, enabling a greater number of plants to be analysed within the same time frame and supporting studies that require statistically relevant sample sizes. The ability of our apparatus to scan both treated and untreated plants within a single 24-hour cycle therefore represents a practical advantage. This capacity enables more reliable comparisons and more effective detection of phenotypic differences, which is particularly valuable in studies investigating the effects of biostimulants or environmental stress factors on plant phenotypes. Taken together, these advantages show that the scanning photogrammetric apparatus described in this study, when properly configured, expands the capabilities of standard laboratory-scale equipment [77, 78].

## Conclusions

This study aimed to develop a non-destructive and cost-effective method for analysing morphological traits in the phenotyping of economically significant plants. The feasibility of a close-range photogrammetry setup utilising structure-from-motion and multi-view stereo techniques was evaluated. For this purpose, an existing robotic scanning system based on a static-camera and moving-object configuration was upgraded. An algorithm enabling automated camera positioning, data acquisition, subsequent processing, and model generation was successfully implemented. The integration of a robotic arm and turntable makes the system robust and provides flexibility in arranging the spatial geometry for photogrammetric reconstruction.

A comprehensive evaluation of the influence of individual parameters on model quality was conducted using both qualitative and statistical indicators. Among all calibration strategies tested, the most accurate results were achieved with simultaneous calibration using pre-calibrated interior orientation parameters combined with adaptive camera model fitting. This configuration significantly improved spatial reconstruction quality. Other approaches, particularly those based only on focal length and pixel size, reduced accuracy or led to partial alignment failure. These findings confirm the critical role of camera calibration in ensuring reconstruction reliability, especially when non-metric cameras are used. The optimal settings were consistent for all species of plants tested. The optimal exposure time was 50 milliseconds, and the ideal camera-to-object distance was 16 centimetres. The most reliable depth-map-based reconstructions were achieved when the tweak parameter was set to 0.9, whereas leaving the tweak unset resulted in poor model quality. A configuration characterised by a reduced number of images combined with an increased number of height levels was found to contribute to the generation of higher-quality 3D models of plants compared to a configuration with a less robust image configuration and a greater number of images. The best combination was determined to be three height levels (P1, P3 and P5), with 40 images per position and approximately 10° spacing. The overall evaluation process was successfully streamlined and the time required to process the acquired data was reduced by approximately 75%, lowering the evaluation time from 8 min to only 2.7 min per plant while accuracy was maintained. Most vertices within the meshes exhibited confidence levels approaching 100%.

This methodology expands the range of photogrammetric analysis-based phenotyping platforms. The apparatus combines low-cost, reliability, ease of use, accuracy, robotic-assisted flexibility, and time efficiency. Importantly, the favourable balance between scanning speed and reconstruction quality shows that efficiency does not compromise detailed 3D modelling. The system can be easily adapted beyond the current limit of 30–40 cm by simple structural modifications. In general, it represents a competitive platform for high-throughput plant phenotyping. This platform has promising applications in the development and optimisation of biopreparations that enhance plant growth, vitality, and resilience in sustainable agriculture.

## Data Availability

No datasets were generated or analysed during the current study.
